# Persian Translation and Cultural Adaptation of Mini International Neuropsychiatric Interview and its Psychometric Properties

**DOI:** 10.34172/aim.2022.48

**Published:** 2022-05-01

**Authors:** Ali-Akbar Nejatisafa, Elham Sharafi, Mahtab Motamed, Atefeh Mohammadjafari, Farnaz Etesam, Nazila Shahmansouri, Mohammad Arbabi, Elham Haki-Kazazi, Hamideh Sadrameli, Mahdi Hormozpoor, Ahmad Ali Noorbala

**Affiliations:** ^1^Psychosomatic Research Center, Department of Psychiatry, Tehran University of Medical Sciences, Tehran, Iran; ^2^Research Center for Cognitive and Behavioral Sciences, Tehran University of Medical Sciences, Tehran, Iran

**Keywords:** Diagnosis, Interviews, Iran, Psychiatry, Psychometrics

## Abstract

**Background:**

The main objectives of this study were the translation, cultural adaptation, and assessment of the psychometric properties of the Persian version of Mini International Neuropsychiatric Interview (MINI).

**Methods:**

All processes of linguistic methodology were conducted according to the published guidelines. A total of 180 patients with psychiatric problems were interviewed using MINI and Structured Clinical Interview for DSM-5(R) - Clinician Version (SCID-5-CV) by different interviewers. Another 30 patients were selected for examining the test-retest reliability. The study sample was recruited from a psychiatric hospital and a general hospital in Tehran, Iran. Face validity, feasibility, time of the interview, test-retest reliability, and concurrent validity were evaluated.

**Results:**

Mean interview time was 19.76±10.30 minutes, indicating satisfactory feasibility. The test-retest reliability was very good (phi=2, Cramer’s V=0.89, *P*<0.0001). The kappa values showed good or excellent agreement between MINI and SCID-5-CV for psychotic disorders (0.88), substance-related disorders (0.86), bipolar disorder (0.85), major depressive disorder (0.84), obsessive-compulsive disorder (0.74), and mental disorder due to other medical disorders (0.7). However, the kappa values were found to be lower for generalized anxiety disorder (0.44) and posttraumatic stress disorder (0.32) diagnoses.

**Conclusion:**

The Persian version of MINI is a feasible, reliable, and valid instrument for diagnosing some mental disorders. Further research is needed to evaluate the validity of this instrument in other categories of psychiatric diagnoses in the general population.

## Introduction

 The importance of accurate diagnoses to provide patients with best practice and care is undeniable. In the lack of objective para-clinic testing, diagnostic clinical interviews have remained as gold standards in most psychiatric diagnoses. Standard diagnostic interviews have been developed to provide structure to clinical questions and enhance the process of diagnosis. However, researchers usually argue that the implementation of comprehensive diagnostic interviews needs a long time and costs. The most widely known structured interviews, namely, the Structured Clinical Interview for Diagnostic and Statistical Manual for Mental Disorders (SCID),^1^ Composite International Diagnostic Interview for ICD-10 (CIDI),^2^ Schedules for Clinical Assessment in Neuropsychiatry (SCAN)^3^ and Schedule for Affective Disorders and Schizophrenia (SADS)^4^ comprise more than 500 questions and require 1–2 hours to conduct. Prolonged interviews can be hard to conduct, particularly when patients are medically ill and suffering from pain or anticipating a procedure. In addition, it takes quite a considerable time – usually about 5 days to train raters in order to apply the instruments mentioned above.

 The Mini International Neuropsychiatric Interview (MINI) was designed to fulfill the requirement for a short and reliable structured diagnostic interview in clinical as well as research settings.^5,6^ It is a structured assessment instrument for major mental health problems developed by Lecrubier et al and Sheehan et al.^5,6^

 The MINI covers 23 disorders from the tenth revision of the International Classification of Diseases and Related Health Problems (ICD-10)^1^ and the Diagnostic and Statistical Manual of Mental Disorders, Fourth Edition (DSM IV).^2^ The MINI’s shorter length makes it more cost-beneficial to use and easier to be utilized in clinical settings. There are some reports that the MINI is acceptable by patients and interviewers, and general practitioners were also satisfied when using it. According to Pettersson et al, the MINI could be of help as part of the clinical assessment of patients in primary care.^7^ The prevalence of psychiatric disorders is higher in general hospitals than in community settings. There is a requirement for psychiatric diagnostic tools that enable reliable diagnoses in patients with medical/surgical problems who are admitted in general hospitals and have comorbid psychosomatic or psychiatric problems. Moreover, the MINI has been widely used in medical disciplines other than psychiatry. As such, Maizels et al reported the MINI to be well applicable for comprehensive psychiatric assessments in patients with headaches.^8^ If a psychiatric diagnostic instrument is translated into a new language, then its psychometric properties should be reassessed.^9^

 This study aims to develop a Persian version of MINI and assess its reliability and validity in a sample of Iranian patients.

## Materials and Methods

###  Translation and Cultural Adaptation

 All stages of linguistic methodology including translation and cultural adaptation were performed according to the published guidelines by Sousa and Rojjanasrirat.^10^ Therefore, two independent translators have translated the English version of the MINI questionnaire into Persian meticulously. All of the discordances between the two translated versions were discussed and rephrased by the translators until a consensus version was finally developed. A skilled English translator who was blinded to the original version of the MINI back-translated the Persian version into English. Consequently, an expert group discussion was held to reach a consensus about the cultural equivalence between the English and the Persian version of the instrument. This expert group (including five psychiatrists and one psychologist) reviewed both versions of the MINI rigorously and made a few changes to the Persian translation. Finally, as a feasibility study, five patients were interviewed by the Persian version of the MINI to evaluate the comprehensibility and utility of the instrument and to revise it for any unclarity and ambiguity.

###  Instruments

####  Mini International Neuropsychiatric Interview 

 The MINI, which was developed originally in 1990 by Mental health professionals in the United States and Europe, was a brief, structured diagnostic interview for DSM-III-R and ICD-10 psychiatric disorders. Subsequently, newer versions of the instrument were developed in both paper and electronic versions. The MINI consists of different modules recognized by the letters of the alphabet (A to J), each corresponding to a specific diagnostic classification. Module A: major depressive disorder; module B: suicidality; module C: bipolar disorders; module D: panic disorder; module E: agoraphobia; module F: social anxiety disorder; module G: obsessive-compulsive disorder; module H: posttraumatic stress disorder; module I: alcohol use disorder; and module J: substance use disorder (non-alcohol). The MINI needs about 15 minutes for administration; therefore, it can be considered as the diagnostic tool of choice in psychiatric assessment and evaluation of outcomes in most of the studies in the field of clinical psychopharmacology and psychiatric epidemiology. Very good inter-rater and test-retest reliabilities have been reported for the English and French versions,^11^ as well as good to very good concurrent validity against CIDI (kappa values between 0.43 to 0.82)^5,6^ and SCID (kappa values between 0.5 to 0.9).^6^ Kappa values indicated that inter-rater agreement was 75% or above.^5^ The MINI has been translated into more than 40 languages, and its psychometric properties have been assessed for the original English and the French versions as well as the Spanish,^12^ Italian,^13^ Japanese,^14^ Moroccan,^15^ Portuguese,^16^ and Norwegian versions.^17^

####  Structured Clinical Interview for DSM-5(R) - Clinician Version 

 The Structured Clinical Interview for DSM-5(R) - Clinician Version (SCID-5-CV) facilitates clinical diagnosis through a phased process based on DSM-5. Each Interview question corresponds easily with a DSM-5 criterion, which helps the interviewers to determine each as either fulfilled or unfulfilled. The SCID-5-CV is a concise and rearranged variant of the SCID, the most widely used structured diagnostic interview administered in mental health research during the past 30 years. The SCID-5-CV is a distinctive and helpful instrument that includes the most commonly observed DSM-5 diagnoses in clinical situations: mood disorders; schizophrenia spectrum and other psychotic disorders; substance use disorders; anxiety disorders (panic disorder, agoraphobia, social anxiety disorder, generalized anxiety disorder); obsessive-compulsive disorder; posttraumatic stress disorder; attention-deficit/hyperactivity disorder; and adjustment disorder.^18^ We used the Persian version of SCID-5 which was previously prepared by Sharifi et al^19^ The validity and reliability of SCID-IV were evaluated in the previous research.^20^ As the criteria of major psychiatric disorders did not change significantly, we can extrapolate these psychometric properties to the newest version of SCID.

####  Raters

 We chose three raters for MINI and two raters for SCID in Roozbeh Hhospital. In Imam Khomeini hospital we had two raters for MINI and three raters for SCID. All of the raters were board-certified psychiatrists or fellows of psychosomatic medicine. All of the SCID raters were qualified for implementing the instrument as they were trained to use this instrument during their participation in the previous research. For MINI raters, we had both group and individual sessions for training. All of the raters were blinded to the patients’ diagnosis that was mentioned in the patients’ records in the hospital.

###  Patients

 According to the developers of the MINI, it is applicable for assessment of psychiatric patients in the community, general hospitals, and psychiatric hospitals. Therefore, we decided to choose a blended sample of participants both from a general hospital and a psychiatric hospital.

 The participants in the Roozbeh psychiatric hospital were recruited from inpatients who were admitted to the hospital and agreed to participate in the study. The sampling method was convenient sampling. As most of the patients who are admitted to psychiatric wards have acute behavioral problems, we interviewed the patients in the second week of their admission.

 The participants in Imam Khomeini hospital consisted of two groups. The first group, who were chosen to assess the test-retest reliability of the MINI, were recruited from patients admitted to the psychosomatic ward of Imam Khomeini hospital. The second group consisted of outpatients referred to the outpatient psychosomatic clinic of the Imam Hospital.

###  Procedure

 In the first step, the patients were interviewed with MINI, and the demographic characteristics of patients, main diagnosis, and comorbid diagnoses, and the duration of the interview were recorded on a separate sheet. In the second step, all the recruited patients were interviewed again using the SCID with different raters within a week. The main and the comorbid diagnoses were also recorded.

###  Statistical Analyses

 Patient characteristics and demographics were analyzed with descriptive statistics. We used cross-tabulation and Cramer’s V statistics for test-retest reliability. To evaluate validity, we used two indices: simple agreement and Cohen’s kappa. All statistical analyses were performed using IBM-SPSS version 24.

## Results

###  Translation

 Two changes were made during the process of translation. First, in the general instruction part of the original English MINI, the authors have mentioned, “Sentences written in ‘the normal font’ should be read exactly as written to the patient to standardize the assessment of diagnostic criteria. Sentences written in ‘CAPITALS’ should not be read to the patient. These are instructions for the interviewer to assist in the scoring of the diagnostic algorithms.” As we do not have capital letters in the Persian language, we substituted this writing style with a distinguished and different Farsi font in Microsoft Word software. Second, in section J of the MINI interview which pertains to substance use disorder (non-alcohol), we use a local common name for different substances. For example, “crack” in Iran is the common name for crystallized heroine, which in a Western country, is the name of a special kind of cocaine. Furthermore, in some instances, the brand names of sedative medications were used in the English version that was substituted with the generic name of these medications. For example, we used “Lorazepam” instead of “Ativan.”

###  Duration of the Interview with MINI

 The minimum and maximum duration of the interview were 9 and 30 minutes, respectively. The mean ± SD for the duration of interviews was 19.76 ± 10.30 minutes.

###  Patients Characteristics

 Among 190 patients approached for the interview, 170 patients accepted to participate and complete the interview. The final sample consisted of 150 patients from Roozbeh hospital and 20 patients from Imam Khomeini hospital outpatient clinic. In addition, for evaluation of test-retest reliability, we included 30 other inpatients from the psychosomatic ward of Imam Khomeini hospital. Tables 1 and 2 show the demographics of the main sample and the test-retest sample, respectively.

**Table 1:** Demographic Characteristics of the Main Sample of the Study (n = 180)

**Variables**	
Gender, No. (%)	
Female	76 (44.70)
Male	94 (55.30)
Age, Mean ± SD	37.70 ± 12.08
Marital status, No. (%)	
Married	68 (40)
Single	80 (47.05)
Widowed	4 (2.35)
Divorced	18 (10.60)
Education, No. (%)	
Less than diploma	86 (50.58)
Diploma/Associate degree	62 (36.47)
Bachelor degree/ Master	20 (11.77)
PhD or higher	2 (1.18)
Occupation, No. (%)	
Self-employed	40 (23.52)
Homemaker	44 (25.88)
Employer	12(7.05)
Unemployed	64 (37.65)
Student	4 (2.35)
Retired	6 (3.52)
Hospital, No. (%)	
Roozbeh	150 (88.23)
Imam Khomeini	20 (11.76)
Place of residence, No. (%)	
Tehran	117 (68.82)
Other cities	53 (31.18)

**Table 2:** Demographic Characteristics of the Sample for the Test-Restest Assessment Part of the Study (n = 30)

**Variables**	
Gender, No. (%)	
Female	17 (56.66)
Male	13 (43.34)
Age, Mean ± SD	41.16 ± 14.13
Marital status, No. (%)	
Married	16 (53.33)
Single	9 (30)
Widowed	2 (6.67)
Divorced	3 (10)
Education, No. (%)	
Less than diploma	9 (30)
Diploma/Associate degree	17 (56.66)
Bachelor degree/ Master	4 (13.34)
PhD or higher	0 (0)
Occupation, No. (%)	
Self-employed	7 (23.33)
Home maker	13 (43.33)
Employer	2 (6.67)
Unemployed	6 (20)
Student	0 (0)
Retired	2 (6.67)
Hospital, No. (%)	
Roozbeh	0 (0)
Imam Khomeini	30 (100)
Place of residence, No. (%)	
Tehran	23 (76.66)
Other Cities	7 (23.34)

###  Assessment of the Test-Retest Reliability of MINI

 We used Cramer’s V for statistical analysis, as the rating of patients repeated within 10-day periods by a single rater, and the variable was nominal. Table 3 shows the test and retest diagnosis of patients who were assessed in the test-retest procedure. The test-retest reliability was very good (phi = 2, Cramer’s V = 0.89, *P* < 0.0001).

**Table 3:** Cross-tabulation of Test and Retest Diagnoses According to MINI

**MINI-Retest Diagnosis**											
**MINI-Test Diagnosis**		**Agoraphobia**	**Bipolar D**	**GAD**	**MDD**	**OCD**	**Panic D**	**Psychotic D**	**PTSD**	**SAD**	**Total (AP)**
	Bipolar D	0	5	0	1	0	0	0	0	0	6 (83.33)
	GAD	0	0	1	0	0	0	0	0	1	2 (50)
	MDD	0	1	2	10	1	0	0	0	0	14 (92.85)
	OCD	1	0	0	0	1	0	0	1	0	3 (66.66)
	Panic D	0	0	0	0	0	1	0	0	0	1 (100)
	Psychotic D	0	0	0	0	0	0	4	0	0	4 (100)
	Total (AP)	1 (0)	6 (83.33)	3 (66.66)	11 (72.72)	2 (50)	1 (100)	4 (100)	1 (0)	1 (0)	30 (83.33)

MINI, Mini International Neuropsychiatric Interview; Bipolar D, bipolar disorder; GAD, generalized anxiety disorder; MDD, major depressive disorder; OCD, obsessive-compulsive disorder; Panic D, panic disorder; Psychotic D, psychotic disorder; PTSD, post-traumatic stress disorder; SAD, social anxiety disorder; AP, agreement percentage.

###  Assessment of Validity of MINI (Agreement with SCID-5-CV)

 For assessment of validity, simple agreement and Cohen’s kappa between MINI and SCID-5-CV interview were calculated. The best agreement was found for patients with the diagnosis of any psychotic disorders followed by substance-related disorders and bipolar disorder with kappa coefficients of 0.88, 0.86, and 0.85, respectively. Kappa values indicated excellent agreement (more than 0.75) for any psychotic disorders followed by substance-related disorders, bipolar disorder, and major depressive disorder. They revealed good agreement (0.60–0.74) for obsessive-compulsive disorder and mental disorder due to another medical disorder. We found the lowest kappa values for generalized anxiety disorder (0.44) and posttraumatic stress disorder (0.32) (Table 4).

**Table 4:** Concordance Between MINI and SCID-5-CV Diagnoses

**Disorders (** ***n*** ** = 170)**	**MINI**	**SCID**		**Simple agreement**	**Cohen’s Kappa (95% CI)**
		**+**	**-**		
		**+ TP**	** FP**		
		**- FN**	**TN**		
Psychotic Disorders		37	4	95.88	0.88 (0.77–0.99)
		3	136		
Substance-Related Disorders		20	2	98.23	0.86 (0.74–0.96)
		1	157		
Bipolar Disorder		68	7	92.94	0.85 (0.76–0.94)
		5	90		
Major Depressive Disorder		20	7	96.47	0.84 (0.70–0.92)
		5	144		
Obsessive-Compulsive Disorder		3	1	98.82	0.74 (0.38–0.98)
		1	165		
Due to Other Medical Condition		5	3	97.64	0.70 (0.36–0.89)
		1	161		
Post-traumatic Stress Disorder		1	3	97.64	0.32 (0.14–0.92)
		1	165		

SCID, Structured Clinical Interview for DSM-5; MINI, Mini International Neuropsychiatric Interview; TP, true positive; FP, false positive; FN, false negative; TN, true negative.

## Discussion

 In this study, MINI was translated into Persian with meticulous methodology regarding cultural adaptation. In addition, some of the psychometric properties of the Persian version of MINI were evaluated. The results of the study indicated good results for feasibility, test-retest reliability, and validity. For most of the diagnoses, the kappa values were excellent to good including psychotic disorders, substance-related disorders, bipolar disorder, major depressive disorder, obsessive-compulsive disorder, and mental disorder due to other medical disorders. However, the kappa values were lower for generalized anxiety disorder and posttraumatic stress disorder. The average duration of the interview was satisfactory, although longer than the instructions expect.

 The present study’s findings were comparable to previous studies that were performed by the English version and other languages translation. As such, in a study conducted by Kadri et al, the Arabic version of MINI had a good concordance with the experts’ diagnosis, with kappa values greater than 0.8.^15^ In the current study, the agreement for diagnoses like generalized anxiety disorder and posttraumatic stress disorder was lower than other diagnoses. These findings are in line with the studies performed by research groups in Japan and Norway.^14,15^

 The test-retest reliability of the Persian version of MINI was very good. Previous studies also showed that test-retest reliability had excellent results with MINI.^15,16^

 The duration of the interview was about 20 minutes on average^12^ however, this depends on both the interviewer and patient characteristics. Specifically, the interviewer’s experience with MINI may reduce the time to perform the interview.^15^

 The variety in our sampling setting can be regarded as our study strength, since the participants were recruited from two hospitals and both outpatients and inpatients were included. This study also had several limitations. Although the overall sample size was relatively acceptable, the sample size for some diagnoses like anxiety disorders was not enough. Unfortunately, we did not have any patients in diagnostic categories like alcohol-related disorders, eating disorders, and antisocial personality disorder which are included in the MINI. The small sample size did not allow us to differentiate between current and lifetime diagnoses or between subcategories of bipolar disorder and substance-related disorder.

 In conclusion, our data suggest that the Persian version of the MINI had good feasibility, reliability, and validity in comparison with SCID-5-CV in the psychiatric outpatients and inpatients. Further studies with enough sample size for each diagnosis and with non-clinical samples and normal controls are needed in the future.

## References

[1] World Health Organization. The ICD-10 Classification of Mental and Behavioural Disorders: diagnostic criteria for research. Geneva: World Health Organization; 1993.

[2] American Psychiatric Association: Diagnostic and Statistical Manual of Mental Disorders, Fourth edition (DSM-IV). Washington, DC: American Psychiatric Association; 1994.

[3] World Health Organization. Schedules for Clinical Assessment in Neuropsychiatry (SCAN). Geneva: World Health Organization; 1992.

[4] Endicott J, Spitzer RL (1978). A diagnostic interview: the schedule for affective disorders and schizophrenia. Arch Gen Psychiatry.

[5] Lecrubier Y, Sheehan DV, Weiller E, Amorim P, Bonora I, Harnett Sheehan K (1997). The Mini International Neuropsychiatric Interview (MINI): a short diagnostic structured interview, reliability and validity according to the CIDI. Eur Psychiatry.

[6] Sheehan DV, Lecrubier Y, Harnett Sheehan K, Janavs J, Weiller E, Keskiner A (1997). The validity of the Mini International Neuropsychiatric Interview (M.I.N.I.) according to the SCID-P and its reliability. Eur Psychiatry.

[7] Pettersson A, Modin S, Wahlström R, Af Winklerfelt Hammarberg, S S, Krakau I (2018). The Mini-International Neuropsychiatric Interview is useful and well accepted as part of the clinical assessment for depression and anxiety in primary care: a mixed-methods study. BMC Fam Pract.

[8] Maizels M, Smitherman TA, Penzien DB (2006). A review of screening tools for psychiatric comorbidity in headache patients. Headache.

[9] Sartorius N, Janca A (1996). Psychiatric assessment instruments developed by the World Health Organization. Soc Psychiatry Psychiatr Epidemiol.

[10] Sousa VD, Rojjanasrirat W. Translation, adaptation and validation of instruments or scales for use in cross‐cultural health care research: a clear and user‐friendly guideline J Eval Clin Pract 2011;17(2):268-74. 10.1111/j.1365-2753.2010.01434.x 20874835

[11] Shrout PE, Spitzer RL, Fleiss JL (1987). Quantification of agreement in psychiatric diagnosis revisited. Arch Gen Psychiatry.

[12] Bobes J (1998). A Spanish validation study of the mini international neuropsychiatric interview. Eur Psychiatry.

[13] Rossi A, Alberio R, Porta A, Sandri M, Tansella M, Amaddeo F (2004). The reliability of the Mini-International Neuropsychiatric Interview: Italian version. J Clin Psychopharmacol.

[14] Otsubo T, Tanaka K, Koda R, Shinoda J, Sano N, Tanaka S (2005). Reliability and validity of the Japanese version of the Mini-International Neuropsychiatric Interview. Psychiatry Clin Neurosci.

[15] Kadri N, Agoub M, El Gnaoui S, Alami KM, Hergueta T, Moussaoui D (2005). Moroccan colloquial Arabic version of the Mini International Neuropsychiatric Interview (MINI): qualitative and quantitative validation. Eur Psychiatry.

[16] de Azevedo Marques JM, Zuardi AW: Validity and applicability of the Mini International Neuropsychiatric Interview administered by family medicine residents in primary health care in Brazil. Gen Hosp Psychiatry 2008;30(4):303-10. 10.1016/j.genhosppsych.2008.02.001 18585532

[17] Mordal J, Gundersen Ø, Bramness JG: Norwegian version of the MINI-International Neuropsychiatric Interview: feasibility, acceptability and test-retest reliability in an acute psychiatric ward. Eur Psychiatry 2010;25(3):172-7. 10.1016/j.eurpsy.2009.02.004 19553089

[18] First MB WJ, Karg RS, Spitzer RL. Structured Clinical Interview for DSM-5 Disorders, Clinician Version (SCID-5-CV). Arlington, VA: American Psychiatric Association; 2016.

[19] Sharifi V, Shadloo B, Shahrivar Z. Structured clinical interview for DSM-5 disorders [Persian]. Tehran; Ibn-e-Sina; 2019.

[20] Sharifi V, Asadi SM, Mohammadi MR, Amini H, Kaviani H, Semnani Y (2004). Reliability and feasibility of the Persian version of the structured diagnostic interview for DSM-IV (SCID). Advances in Cognitive Science.

